# Autonomy Mediates the Relationship between Personality and Physical Activity: An Application of Self-Determination Theory

**DOI:** 10.3390/sports4020025

**Published:** 2016-04-29

**Authors:** Meredith L. Ramsey, Eric E. Hall

**Affiliations:** Department of Exercise Science, Elon University, Elon, NC 27244, USA; mramsey3@elon.edu

**Keywords:** personality, physical activity, autonomy, self-determination

## Abstract

This study sought to examine tenets of Self-Determination Theory by testing a mediation model of physical activity and personality via autonomy. A total of 290 adults were recruited to complete a one-time online survey of exercise habits and individual characteristics. Surveys assessed personality, autonomy, and physical activity. A measurement model specifying direct effects between personality dimensions and physical activity and indirect effects operating through autonomy provided an excellent fit to the data (Χ^2^ = 0.66, *df* = 3, *p* = 0.88, RMSEA(90% CI) = 0.00 (0.00–0.05), CFI = 0.99, SRMR = 0.01). Results indicated significant (*p* < 0.05) effects of Extroversion (β = 0.42), Conscientiousness (β = 0.96), and Emotional Stability (β = 0.60) on autonomy, which in turn, was significantly associated with physical activity (β = 0.55). No significant effects were observed for Agreeableness or Intellect. None of the personality constructs were found to be directly associated with physical activity. This model accounted for 27% of the variance in physical activity. The results of this study suggest that autonomy is significantly associated with physical activity. Therefore, attempts to improve autonomy in individuals may be a useful intervention strategy in improving physical activity levels.

## 1. Introduction

Despite recent and widespread public health initiatives to increase physical activity, physical inactivity continues to plague today’s society [1,2]. The American College of Sports Medicine recommends that adults should engage in at least 150 min of moderate-intensity exercise each week for cardiorespiratory fitness. This can be accomplished by engaging in moderate-intensity exercise for 30 to 60 min 5 days per week or vigorous-intensity exercise 20 to 60 min 3 days per week [3]. Currently only 43.5% of American adults meet this requirement [1]. Living a sedentary lifestyle has shown many negative health implications including increased risk of cardiovascular disease, metabolic disorders, musculoskeletal disorders, psychological disorders, pulmonary diseases, and some cancers [2].

One theory that has been used to examine motivations and their relationship with physical activity behavior is Self-Determination Theory (SDT) [4]. SDT helps to explain affective, cognitive, and behavioral response within an achievement activity and has been used in multiple areas such as academics and athletics; with more recent efforts to examine these principles within the exercise domain [4,5]. SDT suggests that motivation lies on a continuum ranging from amotivation to intrinsic motivation. Amotivation is described as having no motivation to engage in an activity while intrinsic motivation is when an activity is not done for reward or any form of reinforcement, but done for fun, enjoyment or pleasure [5]. Extrinsic motivation falls between the two and is done because the person expects some sort of reward or outcome [5]. When moving across the continuum from amotivation to intrinsic motivation there is a growing amount of autonomy or self-determination [4]. A recent systematic review found there is positive relationship between more autonomous forms of motivation and physical activity behavior and adherence [6]. These consistent findings have led to theory based interventions, using SDT, to improve autonomy and promote physical activity [7].

In addition to SDT, previous research has examined the relationship between personality and physical activity and found that extraversion, conscientiousness, and neuroticism of the five factor model [8,9] are related to exercise behaviors [10,11,12]. A recent meta-analysis by Wilson and Dishman [13] utilizing 64 studies found small, significant correlations for extraversion, neuroticism and conscientiousness similar to the previous studies mentioned, but also found a relationship with openness. The findings that extraversion and neuroticism are related to physical activity level is consistent with Eysenck’s theory of personality which suggests that extraverts will seek out sensory stimulation (e.g., exercise) and people high in neuroticism may avoid stimulation because of higher levels of negative affect and heightened autonomic response [14]. It is possible that conscientiousness is related to physical activity because it may help the individual satisfy their need for competence and self-regulation [15].

Only two known studies have attempted to examine both SDT and personality as they related to physical activity [15,16]. Ingledew and colleagues [15] examined personality and self-determination in relation to exercise for individuals in the maintenance stage of change. The results indicated participants who were less neurotic, more extroverted, and more conscientious were more likely to be self-determined (higher autonomy) in regard to exercise. A limitation of this study was that they did not try to predict physical activity behavior based on the measures of autonomy and personality, and they only used individuals in the maintenance stage in their data analysis. Furthermore, a study conducted by Lewis and Sutton [16] sought to determine the relationship between personality and exercise participation, while also trying to determine if the relationship was mediated by autonomy. The results indicated that more autonomous behavior was positively correlated with exercise participation. Also, extroversion, conscientiousness, and agreeableness were positively related to increased exercise frequency with extroversion and conscientiousness being mediated by intrinsic and external motivation [16].

The purpose of the current study was to determine the relationship between personality, autonomy, and physical activity behavior. It was hypothesized that personality and autonomy would be directly related to physical activity, but the relationships between personality and physical activity may be mediated by autonomy. It is important to examine mediating pathways through autonomy to identify potential mechanisms as well as target points for intervention, autonomy provides a modifiable pathway by which physical activity levels might be impacted in the presence of certain personality traits.

## 2. Methods

### 2.1. Participants 

Two hundred ninety adults were recruited for this study (218 females, 64 males, 8 non-reported; mean age 23.9 ± 9.2 years; mean BMI 23.8 ± 5.0; 91.7% Caucasian). Participants were recruited through postings on the university website as well as through social media postings by the authors. The study was approved by the university’s IRB and all participants read and consented to participate in the study.

### 2.2. Measures 

The International Personality Item Pool (IPIP) [17] was used to assess personality based on dimensions of extraversion, agreeableness, conscientiousness, emotional stability (opposite of neuroticism), and intellect/imagination [18,19]. There were a total of 50 questions with 10 questions being for each sub-scale and Previous research has found scales generated from the IPIP to be valid and reliable [18,19].

The Behavioral Regulation in Exercise Questionnaire-2 (BREQ-2) [20] was used to measure self-determination and is based on five dimensions including amotivation, external regulation, introjected regulation, identified regulation, and intrinsic regulation. These items from the BREQ-2 are weighed and then aggregated to determine the Relative Autonomy Index with higher scores representing greater autonomy [20]. The Relative Autonomy Index as derived by BREQ-2 has been previously shown to predict physical activity levels [21].

The Godin Leisure-Time Exercise Questionnaire (GLTEQ) was used to assess physical activity habits over a 7-day period [22]. It asks how often individuals engage in light, moderate, and strenuous activities for at least 15 min or more. From these questions a total met score for the past week was determined.

### 2.3. Procedures

The participants completed a one-time online survey. The survey included the informed consent which they electronically singed as well as the IPIP scale, BREQ-2 and the GLTEQ as well as self-reported demographic questions. At the end of the survey the participants were given the opportunity to enter them into the drawing for a gift card. Participant remuneration was structured such that one out of every 10 participants was randomly selected to receive a $20 gift card.

### 2.4. Statistical Analysis 

**Figure 1:**
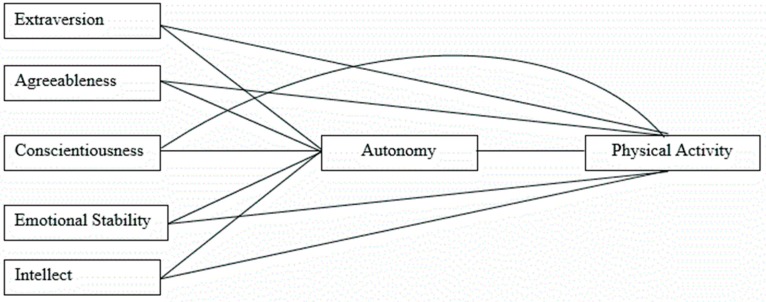
Hypothesized model that was tested with Structural Equation Modeling.

Structural equation modeling (SEM) using MPLUS modeling software (V. 5.1) was used to test the fit of the hypothesized physical activity model (Figure 1). This model specified: (a) direct effects of each of the 5 IPIP sub-scales on physical activity; and (b) indirect effects of the 5 IPIP dimensions on physical activity *through* autonomy. SEM, an extension of general linear modeling, was the preferred analytic method for it allows researchers to test a series of regression equations simultaneously, and unlike regression analysis, does so while accounting for measurement error in the model [23]. Another strength of SEM is that it is designed to test direct and indirect (*i.e.*, mediating) effects among variables. However, SEM has the same limitations of any other type of analyses applied to cross-sectional data, namely that it cannot assess causal relationships in such instances. As SEM requires complete data with no missing values on any variable to conduct model testing, the full-information maximum likelihood (FIML) estimator was used to estimate any missing data on model variables. Using the FIML allowed us to minimize the exclusion of participants from the sample. Of the 290 participants who completed questionnaires, 89% (*n* = 259) provided complete data. 

The fit of the model was tested with several standard goodness of fit criteria; the chi-square statistic, root mean square error of approximation (RMSEA), the comparative fit index (CFI), and the standardized root mean residual (SRMR) [24]. A small chi-square with a nonsignificant *p* value relative to degrees of freedom indicates a good-fitting model. RMSEA reflects the amount of information that is not accounted for by the model. The RMSEA can range from 0 to 1. Values for the RMSEA of .06 or less are indicative of good model fit [24,25]. Similar to RMSEA, the CFI can range from 0 to 1. CFI values of .95 or greater indicate a good model-data fit [24,26]. A value of less than 0.08 for the SRMR indicates an acceptable fit [24].

## 3. Results

**Figure 2:**
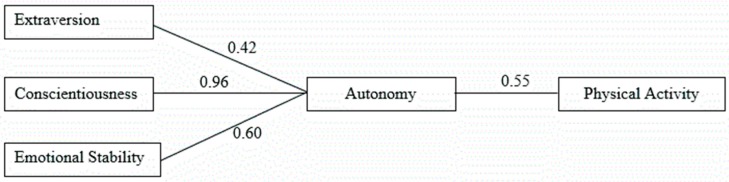
Final Model: only statistically significant (*p* < 0.05) direct paths included in this figure.

Extraversion (β = 0.42), conscientiousness (β = 0.96), and emotional stability (β = 0.60) were significantly associated (*p* < 0.05) with autonomy. No significant effects on autonomy were observed for agreeableness or intellect. As proposed in the theoretical model, autonomy was positively and significantly associated with physical activity (β = 0.55). No significant direct effects on physical activity were observed for any of the personality dimensions. The significant indirect effects of extraversion (β = 0.23), conscientiousness (β = 0.53), and emotional stability (β = 0.33) on physical activity through autonomy provide evidence that autonomy partially mediated this relationship. This model provided an excellent fit to the data (χ^2^ = 2.63, *df* = 5, *p* = 0.76, RMSEA (90% CI) = 0.00 (0.00–0.06), CFI = 0.99, SRMR = 0.01). The final model, shown in Figure 2, accounted for 27% of the variance in physical activity.

## 4. Discussion

The purpose of this study was to determine the relationship between personality, autonomy, and physical activity behavior. It was hypothesized that autonomy as well as the 5 personality domains would all have a direct relationship to physical activity, and that the 5 personality domains would have a direct relationship to autonomy itself (Figure 1). The results indicated that autonomy (β = 0.55) was directly related to physical activity indicating a positive relationship. Additionally, extroversion (β = 0.42), conscientiousness (β = 0.96), and emotional stability (β = 0.60) all showed a direct positive relationship to autonomy and an indirect relationship to physical activity via autonomy. Agreeableness and intellect showed no significant relationship to autonomy, and none of the personality domains showed a significant relationship to physical activity directly. Some of these results are inconsistent with research on personality and exercise behavior which has found extroversion, conscientiousness, neuroticism, and sometimes openness were related to physical activity [10,11,12,13]. The current study showed that these associations were mediated by autonomy. Previous research has found that extraversion and neuroticism have relationships with affect and this may be one potential way that personality and autonomy may work together to influence physical activity levels [27,28]. Additionally, the self-regulation aspect of conscientiousness may help explain its mediation of autonomy and physical activity [15].

The results of the current study were somewhat consistent with the results of the Ingledew and colleagues [15], which found that individuals who were less neurotic and more extroverted and conscientious had a higher relation to self-determination in regard to exercise. These are consistent with the results of the current study in that extroversion, conscientiousness, and emotional stability (the opposite of neuroticism) were positively correlated to autonomy. The results of the present study went one step further in indicating that autonomy was related to reported exercise participation. This is consistent with the results from the systematic review by Teixeira and colleagues [6] which have shown greater autonomy to be related to physical activity behavior. Moreover, the results of the current study were partially supported by the work of Lewis and Sutton [16], who also found that extroversion and conscientiousness were positively related to exercise frequency and were mediated by self-determination. This is consistent with the current results, but the present study also found an indirect relationship between emotional stability and physical activity behavior. Additionally, Lewis and Sutton [16] found a positive relationship between agreeableness and exercise frequency, which the current study did not.

Understanding the influence of personality on autonomy and physical activity may have unique influences on designing effective physical activity interventions. For persons high in conscientiousness the way to improve autonomy may be through setting proper individualized goals. For those high in extraversion emphasizing possible social interaction in group exercise settings may be helpful for them to find enjoyment in physical activity. For those high in neuroticism, focusing on possible mood improvement may be beneficial. Additionally, attempting to give all participants control over choice of activity and focusing on the experience as opposed to outcomes such as weight loss may help build autonomy and increase physical activity behavior. 

This study is not without limitations. One limitation of the study was that the participants were mostly younger adults. There were some participants not within this population, but future research that includes a broader sample in order to have a more generalizable sample of the adult population is warranted. Another limitation of this study is the use of self-report and retrospective measures of physical activity as opposed to monitoring current (objective) physical activity behavior. 

## 5. Conclusion

This study showed that individuals who had high levels of autonomy were more likely to have higher levels of physical activity participation. Additionally, this study concluded that the personality domains of extroversion, conscientiousness, and emotional stability were positively related to autonomy, and that autonomy mediated the relationship between these personality dimensions and exercise participation. This suggests that interventions related to modify and strengthen autonomy may be warranted to promote physical activity. As suggested by Teixeira and colleagues [6], interventions that focus on the experiential rewards of exercise (e.g., enjoyment) or having choices of activities may be a powerful way to reframe thoughts about exercise. However, based on the results of the current study, the role of personality in these interventions should be considered as well, for example the positive affective benefits of exercise might be something those high in neuroticism would embrace [27,28].
